# Multi-Agent Dynamic Resource Allocation in 6G in-X Subnetworks with Limited Sensing Information

**DOI:** 10.3390/s22135062

**Published:** 2022-07-05

**Authors:** Ramoni Adeogun, Gilberto Berardinelli

**Affiliations:** Department of Electronic Systems, Aalborg University, 9220 Aalborg, Denmark; gb@es.aau.dk

**Keywords:** 6G, reinforcement learning, in-X subnetworks, resource allocation, Q-learning, industrial control

## Abstract

In this paper, we investigate dynamic resource selection in dense deployments of the recent 6G mobile in-X subnetworks (inXSs). We cast resource selection in inXSs as a multi-objective optimization problem involving maximization of the minimum capacity per inXS while minimizing overhead from intra-subnetwork signaling. Since inXSs are expected to be autonomous, selection decisions are made by each inXS based on its local information without signaling from other inXSs. A multi-agent Q-learning (MAQL) method based on limited sensing information (SI) is then developed, resulting in low intra-subnetwork SI signaling. We further propose a rule-based algorithm termed Q-Heuristics for performing resource selection based on similar limited information as the MAQL method. We perform simulations with a focus on joint channel and transmit power selection. The results indicate that: (1) appropriate settings of Q-learning parameters lead to fast convergence of the MAQL method even with two-level quantization of the SI, and (2) the proposed MAQL approach has significantly better performance and is more robust to sensing and switching delays than the best baseline heuristic. The proposed Q-Heuristic shows similar performance to the baseline greedy method at the 50th percentile of the per-user capacity and slightly better at lower percentiles. The Q-Heuristic method shows high robustness to sensing interval, quantization threshold and switching delay.

## 1. Introduction

Short-range low-power in-X subnetworks (inXSs) [1,2,3] are receiving attention as potential radio concepts for supporting extreme communication requirements, e.g., reliability above 99.99999, up to a 10Gbps data rate and latencies below 100μs. Similar extreme connectivity requirements have also appeared in recent works on visions for 6th generation (6G) networks [4,5]. InXSs are expected to provide seamless support for applications such as industrial control at the sensor–actuator level, intra-vehicle control, in-body networks and intra-avionics communications even in the absence of connectivity from a traditional cellular network [2,6]. Clearly, these applications represent life critical use cases, necessitating the need to guarantee specified communication requirements everywhere. Such use cases can also lead to dense scenarios (e.g., inXSs inside a large number of vehicles at a road intersection), leading to potentially high interference levels, and hence, the need for efficient interference management mechanisms.

Interference management via dynamic allocation (DA) of shared radio resources has been at the forefront of wireless communication research for several years, see, e.g., [7]. Although several techniques for resource allocation have been studied, the extreme requirements as well as the expected ultra-dense deployments of inXSs makes the interference problem more challenging. This has resulted in a number of published works on resource allocation for wireless networks with uncoordinated deployment of short-range subnetworks [8,9]. In [8], distributed heuristic algorithms were evaluated and compared with a centralized graph coloring (CGC) baseline in dense deployments of inXSs. In [9], a supervised learning method for distributed channel allocation is proposed for inXSs. The works so far focus on only channel selection, making their applicability to other resource selection problems such as the joint channel and power and channel aggregation considered in this paper non-trivial. Moreover, the reliance on full sensing information (SI) by these methods imposes significant overhead on required device capabilities (and hence, cost) as well as radio resources for intra-subnetwork signaling.

To overcome these limitations, we conjecture that reinforcement learning (RL) methods [10,11,12] can be developed to perform resource selection, with potential performance improvement over existing approaches even with only quantized information. Moreover, an RL-based method will eliminate the offline data generation requirement for the method in [9]. The idea is to equip each cell with an agent that learns to adapt resource usage to changing interference conditions.

RL-based methods are becoming increasingly popular in radio resource management (RRM) due to their ability to learn complex decision problems, e.g., allocation of multi-dimensional transmission resources [13] in wireless systems. In particular, multi-agent RL (MARL) is quite popular in recent times due to its capability of achieving a potentially optimal distributed intelligent management of resources. The main advantages of MARL include the ability to: (1) support heterogeneous agents with varying requirements, (2) model local interactions among agents, and (3) distribute computation among agents. To this end, there has been an increase in the number of works applying MARL to RRM in different types of wireless systems, e.g., unmanned aerial vehicle (UAV) communication [11], multi-user cellular systems [14], Industry 4.0 device-to-device communication [15], multibeam satellite systems [16], integrated access and backhaul networks [17], non-orthogonal multiple access [18], multi-cell networks [19], and joint scheduling of enhanced mobile broadband and URLLC in 5th generation (5G) systems [20]. Other studies have applied RL to wireless resource allocation in sensor networks for smart agriculture [21], smart ocean federated learning-based IoT networks [15], and distributed antenna systems [22].

While these studies have shown the potential for learning reasonably good solutions to radio resource optimization problems, they have been predominantly based on the assumption of full environment information and some form of information exchange among the agents. These limit their applicability in practical wireless systems where the overhead associated with signaling of information is an important parameter to be kept at the minimum.

We address the problem of fully distributed and dynamic selection of radio resources for downlink transmission by inXSs operating over a finite number of shared frequency channels. Considering the practical constraints (e.g., cost, processing power, etc.) associated to the signaling of sensing data and channel selection decisions between devices and access points in inXSs, we restrict resources for sensing information and decision exchange (SIDE) to only a single bit per channel. The goal is then to develop a distributed learning method for resource selection based on limited sensing data. Although Deep Q-learning (DQN) [17], which relies on Deep Neural Networks (DNNs) to learn the mapping between sensing measurements and resource selection decisions, has been popular owing to its relatively better scalability compared to classical *table-based Q-learning*, the simplicity of the latter makes it attractive for low-cost radio systems. We therefore focus on developing the MAQL method for dynamic resource selection with lookup tables as the policy. This is reasonable in practical wireless systems, since the size of actions and sensing measurements is bounded by the limited available radio resources, making scalability not much of a problem, particularly, in the case of fully distributed implementations involving only local measurements and individual action selection.

In summary, the main contributions of this paper include the following:

- We cast the resource selection task into a non-convex multi-objective optimization problem involving maximization of the sum capacity at each inXS subject to power, transmission bandwidth and signaling overhead constraints.
- We develop a multi-agent Q-learning (MAQL) solution to solve the problem in a fully distributed manner. To limit the overhead associated with intra-subnetwork signalling, we constrained information exchange within each inXS to a 1-bit channel and adopt a two-level (i.e., 0 and 1) quantization of the SI.
- We further develop an alternative heuristic selection method which utilizes similar quantized information as the MAQL. The algorithm termed Q-Heuristic involves the selection of a resource (or resources) randomly either from the list of resources in level 1 or from the list of all resources in case there are no resources in level 1.
- We apply the MAQL method to the problem of joint channel and transmit power selection for mobile 6G in-XSs. We perform simulations in typical industrial factory settings to evaluate performance gains relative to baseline heuristics with full information and the proposed Q-Heuristic. Unlike existing studies on MAQL for wireless resource management; the simulations include evaluation of the impact of delayed sensing information, which may be inevitable in practice. Extensive evaluation of the sensitivity of the proposed methods to the main design parameters including quantization threshold and switching delay is also performed.

The remainder of this paper is organized as follows. The system and channel models as well as a description of the resource allocation problem is presented in Section 2. The proposed MAQL and Q-Heuristic methods are described in Section 3. This is followed by performance evaluation in Section 4. Conclusions are finally drawn in Section 5.

## 2. System Model and Problem Formulation

**Figure 1:**
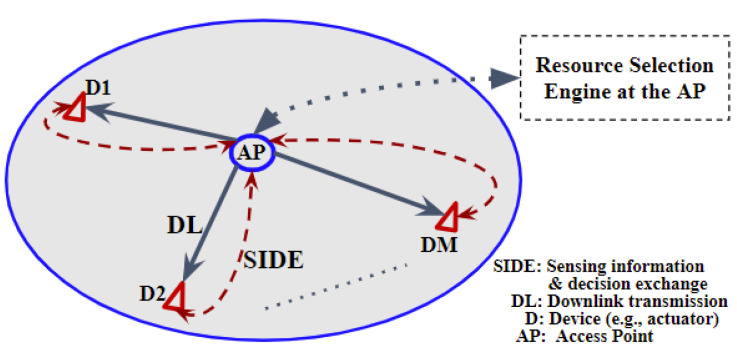
Illustration of DL transmission, and sensing information and resource selection decision exchange in a single inXS.

We consider the downlink (DL) of a wireless network with *N* independent and mobile inXSs each serving one or more devices (including sensors and actuators). The set of all inXSs in the network and the $M_{n}$ devices in the *n*th inXS are denoted as N={1,⋯,N} and $M_{n}=\left\{1,\cdots ,M_{n}\right\}$, respectively. As illustrated in Figure 1, each inXS is equipped with an access point (AP) which coordinates transmissions with all associated devices. The AP is equipped with a local resource selection engine for making decisions based on local sensing data received from its associated devices via a 1-bit SIDE link, as shown in Figure 1.

The inXSs move following a specified mobility pattern which is determined by the application, e.g., inXSs deployed inside mobile robots for supporting factory operations. At any instant, transmissions within each inXS are performed over one of the *K* (K<<N) shared orthogonal frequency channels denoted as K={1,⋯,K} with a transmit power level within the range, $\left[\kappa_{\min},\kappa_{\max}\right]$, where $\kappa_{\min}$ and $\kappa_{\max}$ are the minimum and maximum allowed transmit power levels, respectively. To simplify the problem, we restrict the possible transmit power to a set of *Z* discrete levels, Z={1,⋯,Z}. We assume that transmissions within each inXS are orthogonal, and hence, there is no intra-subnetwork interference. This assumption is reasonable, since the APs can be designed to allocate orthogonal time-frequency resources to their own devices and have also been made in [1,2].

### 2.1. Channel Model and Rate Expression

The radio channel between the APs and devices in the network is characterized by three components: large scale fading, i.e., path-loss and shadowing, and the small-scale effects. The path-loss on a link from node *A* to node *B* with distance $d_{\mathrm{AB}}$ is defined as $L_{\mathrm{AB}}=c^{2}d_{\mathrm{AB}}^{-\alpha }/16\pi^{2}f^{2}$, where $c\approx 3\times 10^{8}\;{\mathrm{ms}}^{-1}$ is the speed of light, *f* is the carrier frequency and α denotes the path-loss exponent. A correlated log-normal shadowing model based on a 2D Gaussian random field is considered [23]. We compute the shadowing on the link from A to B using

(1) $$X_{\mathrm{AB}}=\ln\left\{\frac{1-e^{\left(-\frac{d_{AB}}{d_{c}}\right)}}{\sqrt{2}\sqrt{1+e^{\left(-\frac{d_{AB}}{d_{c}}\right)}}}\left(S\left(A\right)+S\left(B\right)\right)\right\},$$

where S is a two-dimensional Gaussian random process with exponential covariance function and $d_{c}$ denotes the correlation distance. The small scale fading, *h*, is assumed to be Rayleigh distributed. The Jake’s Doppler model is utilized to capture the temporal correlation of *h* [24].

At a given transmission instant, *t*, the received (or interference) power on the link between any two nodes, e.g., from A to B, is computed as:

(2) $$P_{\mathrm{AB}}\left(\kappa_{A}\left(t\right)\right)=\kappa_{A}\left(t\right)L_{\mathrm{AB}}\left(t\right)X_{\mathrm{AB}}\left(t\right){\left|h_{\mathrm{AB}}\left(t\right)\right|}^{2},$$

where $\kappa_{A}\left(t\right)$ denotes the transmit power (in linear scale) of node A at time *t*. Assuming that the *n*th inXS operates over a frequency channel, $c_{k}:$k∈K at time *t*, the received signal to interference and noise ratio (SINR) from its *m*th device can be expressed as

(3) $$\gamma_{nm}\left(c_{k},\kappa^{k}\left(t\right)\right)=\frac{P_{nm}\left(c_{k},\kappa_{n}^{k}\left(t\right)\right)}{\sum_{i\in I_{k}\left(t\right)}P_{ni}\left(c_{k},\kappa_{i}^{k}\left(t\right)\right)+\sigma_{nm}^{2}\left(t\right)},$$

where $I_{k}\left(t\right)$ and $\kappa^{k}\left(t\right)$ denote the set of devices (or APs) transmitting on channel $c_{k}$ at time *t* and their transmit powers, respectively. The term $\sigma_{nm}^{2}\left(t\right)$ is the receiver noise power calculated as $\sigma_{nm}^{2}\left(t\right)=10^{\left(-174+\mathrm{NF}+10\log_{10}\left(W_{k}\right)\right)}$, where $W_{k}$ denotes the bandwidth of $c_{k}$ and NF is the receiver noise figure. Relying on the Shannon approximation, the achieved capacity can be written as

(4) $$\zeta_{nm}\left(c_{k},t\right)\approx W_{k}\log_{2}\left(1+\gamma_{nm}\left(c_{k},\kappa^{k}\left(t\right)\right)\right).$$

### 2.2. Problem Formulation

In this paper, we consider a resource allocation problem involving a fully distributed joint channel and power selection. This problem can be defined as multi-objective optimization tasks involving the simultaneous maximization of *N* objective functions, one for each inXS. Taking the objective function as the lowest achieved capacity at each inXS (denoted $\zeta_{n}=\min\left({\left\{\zeta_{nm}\right\}}_{m=1}^{M_{n}}\right);\;\forall n\in N$), the problem can, formally, be defined as:

(5) $$\begin{array}{cc} P-I: & \;\max_{c,\kappa }\zeta_{1}\left(c_{1}\left(t\right),\kappa_{1}\left(t\right)\right),\cdots ,\max_{c,\kappa }\zeta_{N}\left(c_{N}\left(t\right),\kappa_{N}\left(t\right)\right)\\ \mathrm{st}: & \;\kappa_{\min}\leq \kappa_{n}\leq \kappa_{\max}\;\mathrm{and}\;\mathrm{BW}\left(c_{k}\right)=W_{k}\;\forall n, \end{array}$$

where $c:=\{c_{n}|n=1,\cdots ,N\}$ and $\kappa :=\{\kappa_{n}|n=1,\cdots ,N\}$ denote the set of channel indices and transmit powers for all inXSs, respectively. The term $\mathrm{BW}(c_{k})$ denotes the bandwidth of channel, $c_{k}$.

The problem in (5) involves the joint optimization of multiple conflicting non-convex objective functions and is typically difficult to solve. The independence of the inXSs and the lack of communication coupled with the desire to minimize overhead due to intra-subnetwork signaling via quantization further aggravate the problem. We present an MAQL method with quantized SI for solving this problem in Section 3. An alternative rule-based solution referred to as Q-Heuristic is also presented.

## 3. Resource Selection with Limited Information

We cast the joint optimization problem in (5) as a Multi-Agent Markov Decision Process (MMDP) [25] described as the tuple {S,A,P,R}, where $S=S_{1}\times \cdots \times S_{N}$ is a set of all possible states for all inXSs referred to as state space, $A=A_{1}\times \cdots \times A_{N}$ is the joint action space containing all possible actions (i.e., the set of all possible combinations of channels and power levels), R denotes the reward signal and P:S×A×S→Δ is the transition function [25], where Δ denotes the set of probability distributions over S.

In the considered MMDP, the goal of the *n*th agent is to find an *optimal* policy, $\pi_{n}^{*}$, which is based solely on its local state and action information, resulting in the so-called Partially Observable MMDP (POMMDP) [26]. Typically, $\pi_{n}^{*}$ is obtained as the policy which maximizes the total reward function [18], i.e.,

(6) $$\pi_{t}^{*}\left(s\right)=\max_{\pi_{t}\left(s\right)\in A}\left\{r_{t}\left(s_{t},\pi_{t}\left(s\right)\right)+\gamma \sum_{s^{\prime }\in S}p\left(s_{t},s^{\prime }\right)\pi_{t+1}^{*}\left(s^{\prime }\right)\right\},$$

where γ:0≤γ≤1 denotes the discount factor. To allow mapping for all possible state–action pairs, an alternative representation, Q(s,a), referred to as the Q-function is commonly used. The Q-function for the *n*th agent is given as [25]

(7) $$Q^{n}\left(s,a\right)=r^{n}\left(s,a\right)+\gamma \max_{a^{\prime }}Q^{n}\left(s^{\prime },a^{\prime }\right).$$

Since each agent has access to only local information, solving (7) results in a local maximum at each subnetwork. We assume that the local maxima on each of the *N* agents’ Q-function is equivalent to the global maximum on the joint Q-function for the entire network, i.e.,

(8) $$\arg\max_{a}Q^{\pi }\left(s,a\right)=\left[\begin{array}{c} \arg\max_{a}Q^{1}\left(s,a\right)\\ \vdots \\ \arg\max_{a}Q^{N}\left(s,a\right) \end{array}\right].$$

According to (8), a solution to the resource selection problem can now be obtained via local optimization at each inXS. MAQL enables a solution of the *N* objectives via the simultaneous interaction of all agents with the environment. The Q-function is iteratively estimated according to Bellman’s equation as [27]

(9) $$\begin{array}{cc} Q^{n}\left(s_{t},a\right) & =\left(1-\alpha \right)Q^{n}\left(s_{t},a\right)+\alpha \left(r(s_{t},a)+\right.\\ \; & \;\left.\gamma \max_{a^{{}^{\prime }}}Q^{n^{\prime }}\left(s_{t+1},a^{{}^{\prime }};\pi \right)\right)\;\forall n, \end{array}$$

where α denotes the learning rate and $r_{n}\left(s_{t},a\right)$ is the instantaneous reward received by the agent for selecting action, a∈A at state $s_{t}\in S$. The policy, π(s,a) corresponds to the conditional probability that action *a* is taken by an agent in state, *s*, and it must therefore satisfy $\sum_{a\in A}\pi \left(s,a\right)=1$.

### 3.1. MAQL Procedure for Dynamic Resource Selection

**Figure 2:**
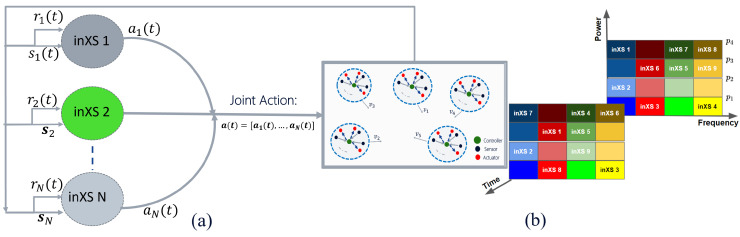
Illustration of the multi-agent RL scenario with N inXSs. (**a**) Multi-agent in-X Subnetwork Scenario; (**b**) Dynamic joint channel and power selections.

To find *optimal* estimates of the Q-functions in (9) via MAQL, we need to define the environment, state space, action space, reward signal, policy representation and training method. As described in Section 2, we consider a wireless environment with *N* independent inXSs each with one or more devices, as illustrated in Figure 2. The remaining components are described below.

#### 3.1.1. State and Observation Space

In the multi-agent scenario, the state of the environment is defined by actions of all inXSs. The achieved performance is also determined by both the *known* local characteristics of each inXS—channel gain, occupied frequency channel, transmit power level, etc., and the *unknown* information about other inXSs. We assume that each inXS has sensing capabilities for obtaining measurements of the aggregate interference power on all channels. This assumption is reasonable, since each inXS device can be equipped with a transceiver that is capable of continuously performing the sensing of its operational channel as well as simultaneously listening on all other channels. We denote the SI at time *t* as $I_{n}^{t}={\left[I_{n,1}^{t},I_{n,2}^{t},\cdots ,I_{n,K}^{t}\right]}^{T}\in R^{\left(K\times 1\right)}$. To account for the effect of channel condition within each inXS, we propose state representation based on the estimated SINR over all channels denoted for the *n*th inXS as $s_{n}^{t}={\left[s_{n,1}^{t},s_{n,2}^{t},\cdots ,s_{n,K}^{t}\right]}^{T}$, with $s_{n,k}=s_{d}/\left(I_{n,k}+\sigma^{2}\right)$, where $s_{d}$ denotes the received signal strength of the weakest link in the inXS. To enable Q-learning, which requires discrete state spaces, we perform a two-level quantization on the SINR, resulting in a state dimension of $\left|S\right|=2^{K}$ comprising all possible combinations of *K* channels each with two levels: 0 and 1. Denoting the SINR quantization value as $s_{\mathrm{th}}$, channel *i* is in state 0 if $s_{n,i}<s_{\mathrm{th}}$ and in state 1 otherwise.

#### 3.1.2. Action Space

For the joint channel and power selection task, the action space is the list of all possible combinations of available frequency channels and transmit power levels in the system. With *K* channels and *Z* discrete power levels, the action selected by inXS *n* at time *t* is from a KZ-dimensional action space comprising all possible combinations of channel and power levels, i.e., $a_{n}^{t}\in A;A=\left\{\left\{c_{1},p_{1}\right\},\left\{c_{1},p_{2}\right\},\cdots ,\left\{c_{K},p_{Z}\right\}\right\}$.

#### 3.1.3. Reward Signal

The reward signal design is a crucial part of the RL design pipeline. This is typically completed by considering the overall goal of the problem and how best to guide an agent toward achieving such a goal. We assume that the communication metric to be maximized is the capacity of the worst link and use (4) as the reward function.

#### 3.1.4. Policy Representation

The decision-making component of any RL method requires a suitable framework for representing what is learnt by an agent during training. This representation is generally referred to as the policy. In this work, the policy at each inXS is represented by a $2^{K}\times \left|A\right|$ *lookup table* containing the Q-values for all state–action pairs. This has the inherent advantage of simplicity and low computation overhead, since decision making is reduced to a simple lookup operation at any given time instant.

#### 3.1.5. Action Selection

Resource selection decision is made by each agent via the ϵ-greedy strategy defined as

(10) $$a_{n}^{t}=\left\{\begin{array}{cc} a\;\mathrm{random}\;\mathrm{selection} & \mathrm{with}\;\mathrm{probability},\;\epsilon \\ \arg\;\max_{a\in A(s_{n}^{t})}Q_{n}\left(s_{n}^{t},a\right), & \mathrm{otherwise} \end{array}\right.,$$

where ϵ is the exploration probability, i.e., the probability that the agent takes random action. During the training, ϵ is decayed at each step according to

(11) $$\epsilon =\max\left(\epsilon_{\min},\left(\epsilon_{\max}-\epsilon_{\min}\right)/\epsilon_{\mathrm{step}}\right),$$

where $\epsilon_{\min}$ and $\epsilon_{\max}$ denote the minimum and maximum exploration probability, respectively, and $\epsilon_{\mathrm{step}}$ is the number of exploration steps.

#### 3.1.6. Training Procedure

Due to its better training stability and fast convergence, a *centralized training with distributed execution* framework which is popular in the multi-agent RL literature is adopted in this paper. A single Q-table is then trained by simultaneously applying it to all inXSs during the training. The procedure is described in Algorithm 1. Once the training is completed, the Q-table is copied to all inXs for fully distributed execution.
**Algorithm 1** Multi-Agent Resource Allocation with Quantized SI: Training Procedure**Input**: Simulation and environment parameters, learning rate, α, discount factor, γ, number of episodes, *T*, number of steps per episode, $N_{e}$, $\epsilon_{\min}$, $\epsilon_{\max}$Start simulator, randomly drop cells and generate shadowing mapt=1; $\epsilon =\epsilon_{\max}$Initialize actions for all cells randomly and compute initial states, ${\left\{s_{n}\left(1\right)\right\}}_{n=1}^{N}$Initialize Q-table, *Q* with zeros**for** t=1**to***T***do**  **for**  i=1 **to** $N_{e}$ **do**    **for**  n=1 **to** *N* **do**      Obtain state from SI $s_{n}\left(t\right)$      Subnetwork *n* select $\;a_{n}\left(t\right)$ according to (10).    **end for**    The joint resource selection of all subnetworks gene-    rate transitions into next states, ${\left\{s_{n}\left(t+1\right)\right\}}_{n=1}^{N}$ and    immediate rewards, ${\left\{r_{n}\left(s\left(t\right),a\right)\right\}}_{n=1}^{N}$    Decay exploration probability using $\epsilon =\max\left(\epsilon_{\min},\left(\epsilon_{\max}-\epsilon_{\min}\right)/\epsilon_{\mathrm{step}}\right)$    **for**  n=1 **to** *N* **do**      Update *Q* using $Q\left(s_{t},a\right)=\left(1-\alpha \right)Q\left(s_{t},a\right)+\alpha \left(r\left(s_{t},a\right)+\gamma \max_{a^{{}^{\prime }}}Q^{{}^{\prime }}\left(s_{t+1},a^{{}^{\prime }};\pi \right)\right)$    **end for**  **end for****end for****Output**: Trained Q-table, *Q*%% The table, *Q* is copied to all APs

### 3.2. Quantized Heuristic

Inspired by our initial results from the MAQL methods, we further proposed the simple *Quantized Heuristic* algorithm for resource selection based on a similar 1-bit SI. The idea is to choose a channel randomly from the list of all channels in the *good* state, i.e., the state with SINR above the quantization threshold, $s_{\mathrm{th}}$. If no channel is in the good state, the channel is chosen randomly from the list of all channels.

## 4. Performance Evaluation

We now train and evaluate the performance of the MAQL approach and compare with fixed (i.e., random assignment at initialization without dynamic updates), greedy channel selection and Q-Heuristic using a snapshot-based procedure. Except where otherwise stated, we consider a network with N=20 inXSs each with a single controller serving as the AP for a sensor–actuator pair in a 50 m × 50 m rectangular deployment area. Each inXS move in the area follows a restricted random waypoint mobility (RRWP) with a constant speed, *v* = 3 m/s. We assume that a total bandwidth B=25 MHz is available in the system and that the bandwidth is partitioned into K=5 channels. Similar to [6,8], we set the transmit power for all inXSs to −10 dBm for all algorithms except MAQL, for which we consider a total of Z=6 transmit power levels between −20 and −10 dBm at intervals of 2 dB, leading to a 30×1 action space. The power difference of ±2 dB is used to ensure reasonable granularity in transmit power levels. Other simulation parameters are shown in Table 1. The deployment and system parameters are defined based on the settings used in [6,8]. The propagation model as well as its parameters are selected from 3GPP documents on channel models for industrial scenarios [28,29].

**Figure 3:**
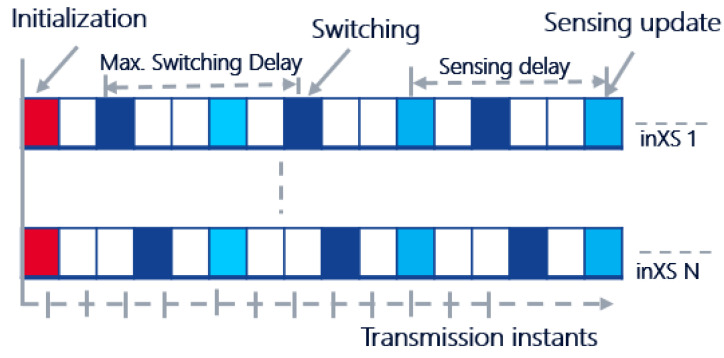
Sensing measurement updates and resource selection with both maximum switching delay and sensing delay equal to 5. InXSs 1 and *N* are assigned random switching integers 2 and 3, respectively. At initialization, all inXs perform random resource selection.

Motivated by the results in [8,9], we introduced random switching delays with a maximum value of $\tau_{\max}=10$ transmission intervals in the simulation. This is to minimize *ping-pong* effects where multiple inXSs simultaneously switch to the same resource. Each inXS is then allowed to switch its operational resource once every 10 transmission instants. The specific time instant at which an inXS has the opportunity to update its transmit power level and/or operational frequency channel is determined by a random integer between 1 and 10. The random integer is assigned to each inXS at the beginning of each snapshot. The concept of switching delay as well as sensing interval is illustrated in Figure 3. Except where stated otherwise, we assume perfect sensing such that measurements for making resource selection and switching decisions are up-to-date with no errors or noise. To understand the impact of imperfect information on achieved performance by the different techniques, we evaluate the algorithms with varying sensing intervals, i.e., time interval between successive update of sensing measurements at each inXS; see the illustration in Figure 3. The results are presented in Section 4.3.

### 4.1. Training, Convergence and Learned Policy

**Figure 4:**
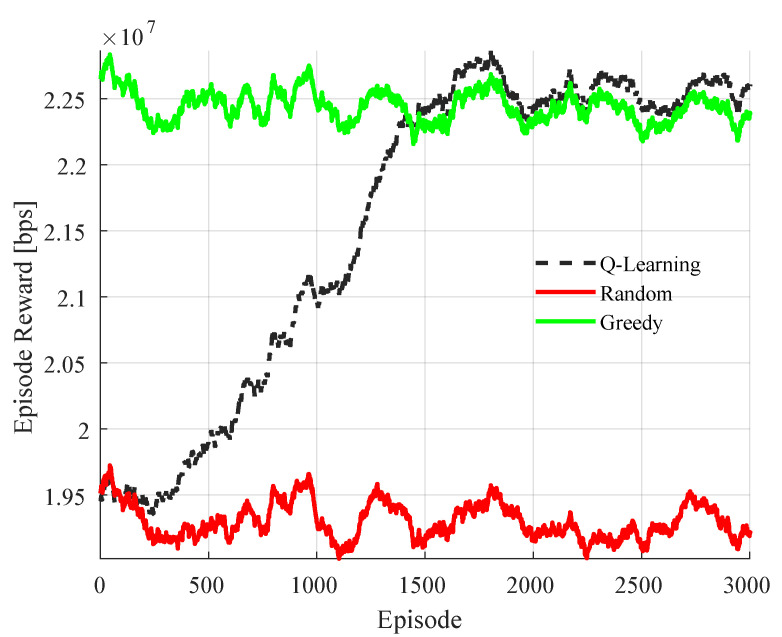
Averaged reward per episode during MAQL training for joint channel and power selection with $s_{\mathrm{th}}=2$ dB.

Figure 4 shows the averaged reward over successive training episodes for the joint power and channel selection problem with SINR quantization threshold, $s_{\mathrm{th}}=2$ dB. The averaging is performed over all steps within each episode as well as all inXSs. We benchmark the reward with those obtained from two heuristic algorithms viz random and greedy channel selection. The maximum transmit power of −10 dBm is used for all inXSs in the heuristic algorithms. The figure shows that the proposed MAQL achieve convergence after approximately 1700 episodes. At convergence, the MAQL method has marginally better performance than the greedy selection baseline with full SI [8].

**Figure 5:**
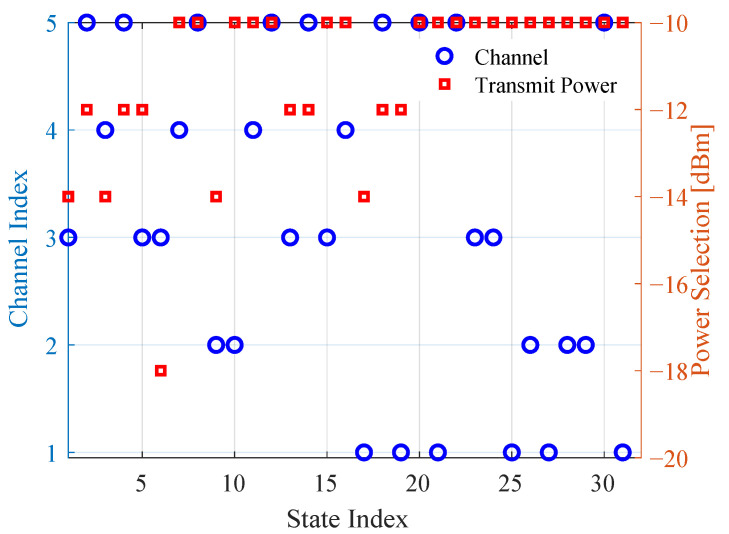
Learned Q-policy at convergence of the MAQL training for joint channel and power selection with $s_{\mathrm{th}}=2$ dB.

To understand the actions of the Q-agents, we show the learned Q-policy at convergence in Figure 5. The policy comprises the channel and transmit power pairs with maximum Q-value at each of the 32 ($2^{5}$) states. The figure shows that the Q-agents converge to a channel with a quantization level of 1 (i.e., with SINR $\geq s_{\mathrm{th}}$) for all states except for state 1, which has no channel in level 1. As shown in the figure, the power levels of −10 dBm, −12 dBm, −14 dBm and −18 dBm are preferred by the agents in the ratio 21:6:4:1. Two power levels, viz, −20 dBm and −16 dBm are never chosen with full exploitation.

### 4.2. Comparison with Benchmark Schemes

**Figure 6:**
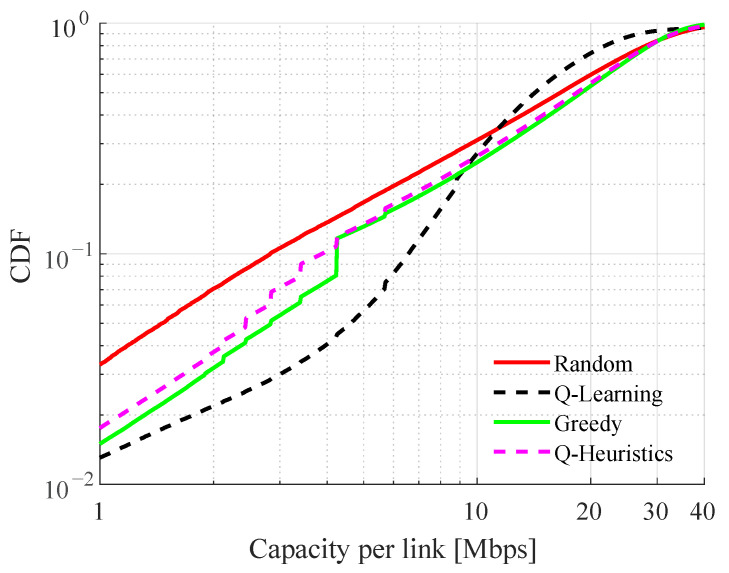
CDF of capacity per inXS with $s_{\mathrm{th}}=2$ dB.

The trained Q-table is deployed at each inXS for distributed resource selection and performance compared with random, greedy channel selection and the proposed Q-Heuristic. Except for MAQL, all algorithms use the maximum transmit power of −10 dBm per transmission as mentioned above. Figure 6 shows the empirical Cumulative Distribution Function (CDF) of the achieved capacity per inXS with sensing-to-action time (i.e., sensing interval) of a single time slot. The proposed MAQL method performs significantly better than simple random selection, Q-Heuristic, and greedy selection with full SI below the 30th percentile of the capacity CDF. This performance improvement appear to have been obtained at the expense of lower capacity above the same percentile. Despite using the same information as MAQL, the Q-Heuristic method is only as good as the greedy baseline. A plausible explanation for the performance improvement by the MAQL is the combined effect of low SINR quantization threshold, $s_{\mathrm{th}}$, and utilization of different power levels.

### 4.3. Sensitivity Analysis

We now present results on sensitivity of the different techniques to quantization threshold, $s_{\mathrm{th}}$, sensing interval, τ, and maximum switching interval.

**Figure 7:**
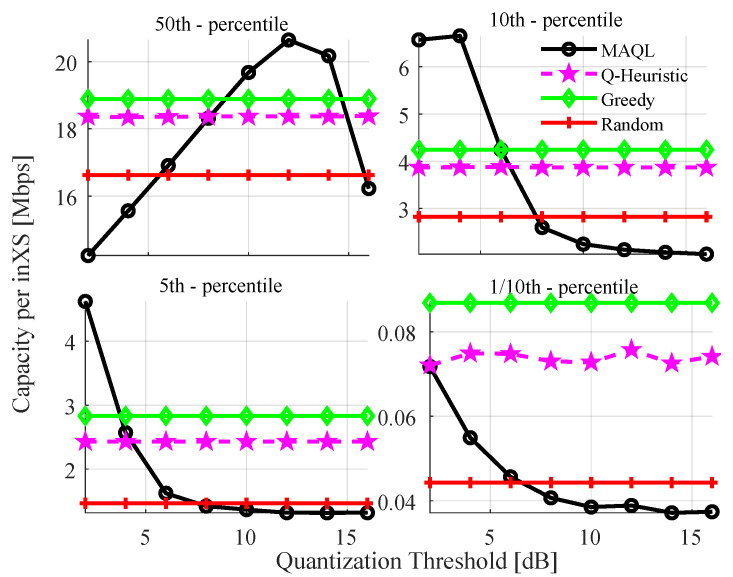
Sensitivity to quantization threshold, $s_{\mathrm{th}}$.

**Figure 8:**
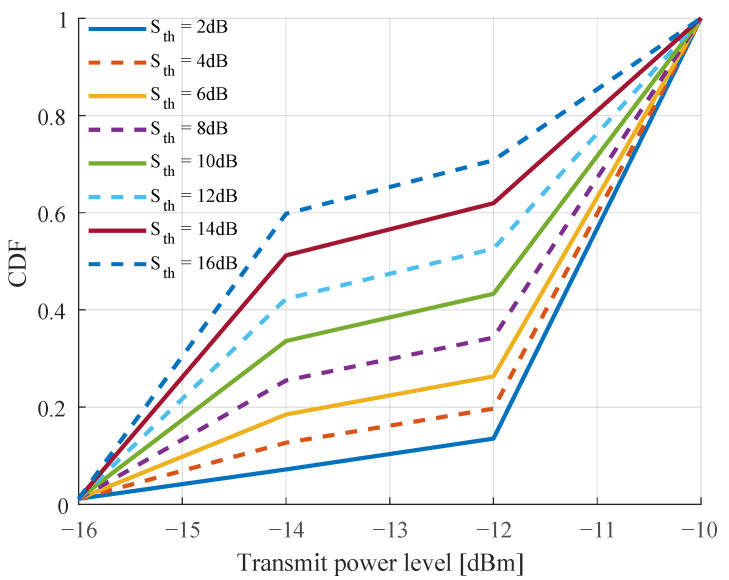
Distribution of selection power by the MAQL method with different $s_{\mathrm{th}}$ values.

In Figure 7, we plot the 50th (median), 10th, 5th and 1/10th percentiles of the capacity per inXS with test quantization thresholds between 2 and 16 dB using the trained policy shown in Figure 5. Note that the training is performed with $s_{\mathrm{th}}=2$ dB. The figure shows that high values of $s_{\mathrm{th}}$ benefit the median of per link capacity while lower values yield higher capacity at the lower percentiles. For instance, the highest 50th, 10th and 5th percentiles of per inXS capacity are achieved with $s_{\mathrm{th}}$ values of 12 dB, 4 dB and 2 dB, respectively. Careful consideration should therefore be taken in setting the threshold based on the communication theoretic targets of the system. In Figure 8, we evaluate the effect of $s_{\mathrm{th}}$ on transmit power selection. The figure indicates that increasing the quantization threshold leads to a higher preference of actions with lower power levels, resulting in a decrease of about ∼3 dB in the median transmit power level with a change in $s_{\mathrm{th}}$ from 2 to 16 dB. A plausible explanation for this trend is that some of the 32 states becomes more likely with increasing (or decreasing) value of $s_{\mathrm{th}}$.

**Figure 9:**
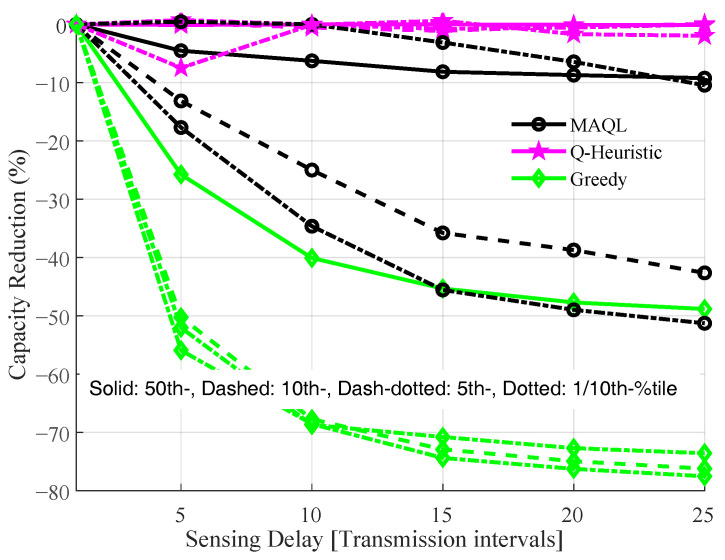
Sensitivity of joint channel and power selection methods to sensing delay, τ with $s_{\mathrm{th}}=2$ dB.

Figure 9 shows the impact of sensing interval on performance of the MAQL, Q-Heuristic and greedy schemes. In this figure, we use the achieved capacity with perfect sensing as a baseline and plot the percentage capacity reduction with increasing sensing interval. The results show that the proposed methods with 1-bit information are in general less sensitive to sensing intervals than the greedy selection method. The Q-Heuristic method exhibits the highest robustness with little or no degradation in capacity with increasing sensing interval. Compared to greedy with up to about 80% capacity decrease, the MAQL has only 50% degradation at a delay of 25 transmission instants. This indicates that the proposed methods offer similar or better performance as the baseline but provide significant overhead reduction for SI exchange as well as better robustness to sensing intervals which may be inevitable in practice.

**Figure 10:**
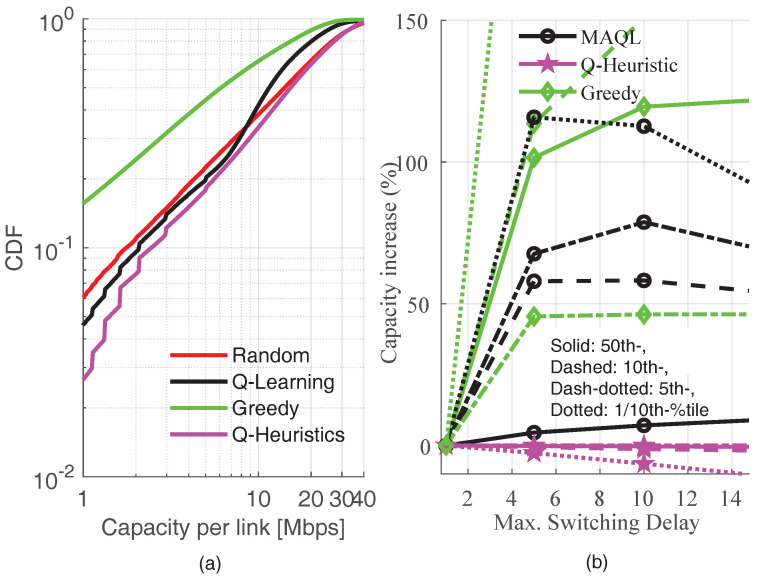
Different percentiles of the capacity per inXS versus maximum switching delay. (**a**) CDF with no switching delay; (**b**) Effect of switching delay.

Finally, we study the effect of switching delay on the performance of the resource selection methods in Figure 10. In Figure 10a, we plot the CDF of capacity per link with maximum switching delay of a single transmission interval. As a result of the simultaneous resource switching and its associated *ping-pong* effects, the greedy algorithm appears to be much worse than all other methods. This indicates that fully greedy resource selection is detrimental to performance in scenarios where controlled switching is not possible. Note that the performance of the MAQL is also degraded in the region below the 30th percentile when compared to Figure 6. To further quantify the effects of switching delay, we plot the capacity increase (in percentage) as a function of the maximum switching delay. The capacity increase at a given maximum delay value is calculated by subtracting the capacity value from its value with no delay. As shown in the figure, it is indeed beneficial to minimize *ping-pong* effects by introducing the switching delay as stated in [9]. Except for the Q-Heuristic which appears to be quite robust to switching delay, a maximum delay of 5 transmission intervals yields capacity increase above 100% for both MAQL and greedy selection methods. As seen in the figure, the greedy method is much more sensitive to switching delays than the proposed MAQL method, which exhibits quite marginal sensitivity at the median of achieved capacity.

We remark here that although the performance evaluation presented in this section is based on 3GPP models for an industrial environment [29], it is often useful to study the sensitivity of the new methods to variations in the wireless environment. For instance, the MAQL method can be evaluated with environment parameters, deployment density and/or configurations that are different from those used during the training, leading to understanding of the ability of the proposed method to generalize to other settings. However, such sensitivity analysis is left for future work. The methods proposed in this paper also consider a single bit per channel which represents the lowest overhead for signaling information about the status of each channel within each inXS. It may then be possible to improve the performance of the proposed schemes with an increased number of bits per channel. Since inXSs are expected to be low-cost radio devices, we believe that the best solutions are those which require minimum signaling overhead without significant performance degradation. Another interesting avenue for further study would be to quantify the trade-off between performance and signaling overhead.

## 5. Conclusions

Multi-agent Q-learning for distributed dynamic resource selection with quantized SI can achieve better performance to the best-known heuristics (i.e., greedy selection) with full information in 6G in-X subnetworks. This is particularly true for the low percentile of the capacity per link and depends on appropriate selection of the value of the SINR quantization threshold, $s_{\mathrm{th}}$. With low $s_{\mathrm{th}}$ values (e.g., between 2 and 4 dB), the MAQL method performs better than both greedy and Q-Heuristic schemes at the 10th, 5th and 1/10th percentiles of per link capacity but worst at the 50th percentile. In contrast, higher $s_{\mathrm{th}}$ values (e.g., between 10 and 14 dB) benefit the 50th percentile of capacity per link but suffers the lower percentiles. Simulation results have shown that the proposed *lookup table*-based MAQL method exhibits fast convergence and is more robust to sensing intervals and switching delays than greedy resource selection. A proposed alternative rule-based scheme based on similar 1-bit SI as the MAQL offers improved robustness with similar performance as the greedy selection baseline. Our ongoing work is investigating other learning-based methods with the capability for optimal performance while eliminating the need for controlled switching via the introduction of switching delays.

## Figures and Tables

**Table 1:** Simulation parameters.

Deployment and System Parameters	
**Parameter**	**Value**
Deployment area (m${}^{2}$)	50 × 50
Number of controllers/inXSs, *N*	20
Number of devices per inXS, *M*	1
Cell radius (m)	3.0
Velocity, *v* (m/s)	3.0
Mobility model	RRWP
Number of channels, *K*	5
**Propagation and Radio Parameters**	
Pathloss exponent, γ	2.2
Shadowing standard deviation, $\sigma_{s}$ (dB)	5.0
De-correlation distance, $d_{c}$ (m)	2
Lowest frequency (GHz)	3
Transmit power levels (dBm)	[−20:2:−10]
Noise figure (dB)	10
Per channel bandwidth (MHz)	5
**Q-Table and Simulation Settings**	
Action space size, |A|	30
Discount factor, γ	0.90
Learning rate, α	0.80
Number of training episodes/steps per episode	3000/200
Minimum/maximum exploration probability	0.01/0.99
Number of epsilon greedy steps	$4.8\times 10^{5}$

## Data Availability

Not applicable.

## References

[1] Berardinelli G., Baracca P., Adeogun R., Khosravirad S., Schaich F., Upadhya K., Li D., Tao T.B., Viswanathan H., Mogensen P.E. (2021). Extreme Communication in 6G: Vision and Challenges for ‘in-X’ Subnetworks. IEEE Open J. Commun. Soc..

[2] Adeogun R., Berardinelli G., Mogensen P.E., Rodriguez I., Razzaghpour M. (2020). Towards 6G in-X Subnetworks With Sub-Millisecond Communication Cycles and Extreme Reliability. IEEE Access.

[3] Berardinelli G., Mahmood N.H., Rodriguez I., Mogensen P. Beyond 5G Wireless IRT for Industry 4.0: Design Principles and Spectrum Aspects. Proceedings of the 2018 IEEE Globecom Workshops (GC Wkshps).

[4] Ziegler V., Viswanathan H., Flinck H., Hoffmann M., Räisänen V., Hätönen K. (2020). 6G Architecture to Connect the Worlds. IEEE Access.

[5] Viswanathan H., Mogensen P.E. (2020). Communications in the 6G Era. IEEE Access.

[6] Adeogun R., Berardinelli G., Mogensen P.E. (2022). Enhanced Interference Management for 6G in-X Subnetworks. IEEE Access.

[7] Hussain F., Hassan S.A., Hussain R., Hossain E. (2020). Machine Learning for Resource Management in Cellular and IoT Networks: Potentials, Current Solutions, and Open Challenges. IEEE Commun. Surv. Tutor..

[8] Adeogun R., Berardinelli G., Rodriguez I., Mogensen P.E. Distributed Dynamic Channel Allocation in 6G in-X Subnetworks for Industrial Automation. Proceedings of the IEEE Globecom Workshops.

[9] Adeogun R.O., Berardinelli G., Mogensen P.E. Learning to Dynamically Allocate Radio Resources in Mobile 6G in-X Subnetworks. Proceedings of the 2021 IEEE 32nd Annual International Symposium on Personal, Indoor and Mobile Radio Communications (PIMRC).

[10] Zhao G., Li Y., Xu C., Han Z., Xing Y., Yu S. (2019). Joint Power Control and Channel Allocation for Interference Mitigation Based on Reinforcement Learning. IEEE Access.

[11] Cui J., Liu Y., Nallanathan A. (2020). Multi-Agent Reinforcement Learning-Based Resource Allocation for UAV Networks. IEEE Trans. Wirel. Commun..

[12] Xiong Z., Zhang Y., Niyato D., Deng R., Wang P., Wang L. (2019). Deep Reinforcement Learning for Mobile 5G and Beyond: Fundamentals, Applications, and Challenges. IEEE Veh. Technol. Mag..

[13] Nguyen T.T., Nguyen N.D., Nahavandi S. (2020). Deep Reinforcement Learning for Multiagent Systems: A Review of Challenges, Solutions, and Applications. IEEE Trans. Cybern..

[14] Meng F., Chen P., Wu L., Cheng J. (2020). Power Allocation in Multi-User Cellular Networks: Deep Reinforcement Learning Approaches. IEEE Trans. Wirel. Commun..

[15] Kwon D., Jeon J., Park S., Kim J., Cho S. (2020). Multi-Agent DDPG-based Deep Learning for Smart Ocean Federated Learning IoT Networks. IEEE Internet Things J..

[16] Liu S., Hu X., Wang W. (2018). Deep Reinforcement Learning Based Dynamic Channel Allocation Algorithm in Multibeam Satellite Systems. IEEE Access.

[17] Lei W., Ye Y., Xiao M. (2020). Deep Reinforcement Learning Based Spectrum Allocation in Integrated Access and Backhaul Networks. IEEE Trans. Cogn. Commun. Netw..

[18] He C., Hu Y., Chen Y., Zeng B. (2019). Joint Power Allocation and Channel Assignment for NOMA With Deep Reinforcement Learning. IEEE J. Sel. Areas Commun..

[19] Ahmed K.I., Tabassum H., Hossain E. (2019). Deep Learning for Radio Resource Allocation in Multi-Cell Networks. IEEE Netw..

[20] Li J., Zhang X. (2020). Deep Reinforcement Learning Based Joint Scheduling of eMBB and URLLC in 5G Networks. IEEE Wirel. Comm. Lett..

[21] Tyagi S.K.S., Mukherjee A., Pokhrel S.R., Hiran K.K. (2020). An Intelligent and Optimal Resource Allocation Approach in Sensor Networks for Smart Agri-IoT. IEEE Sens. J..

[22] Liu Y., He C., Li X., Zhang C., Tian C. (2019). Power Allocation Schemes Based on Machine Learning for Distributed Antenna Systems. IEEE Access.

[23] Lu S., May J., Haines R.J. Effects of Correlated Shadowing Modeling on Performance Evaluation of Wireless Sensor Networks. Proceedings of the 2015 IEEE 82nd Vehicular Technology Conference (VTC2015-Fall).

[24] Jakes W.C., Cox D.C. (1994). Microwave Mobile Communications.

[25] Mukhopadhyay S., Jain B. Multi-agent Markov decision processes with limited agent communication. Proceedings of the 2001 IEEE International Symposium on Intelligent Control (ISIC ’01).

[26] Yang Y., Wang J. (2020). An Overview of Multi-Agent Reinforcement Learning from Game Theoretical Perspective. arXiv.

[27] Sutton R.S., Barto A.G. (2018). Reinforcement Learning: An Introduction.

[28] 3GPP (2018). Study on Communication for Automation in Vertical Domains (Release 16). Technical Report 22.804 v16.2.0, 3rd Generation
Partnership Project. https://www.3gpp.org/ftp/Specs/archive/22\protect\T1\textdollar_\protect\T1\textdollarseries/22.804/.

[29] 3GPP (2020). Study on Channel Model for Frequencies from 0.5 to 100 GHz (Release 16). Technical Report 38.901 v16.1.0, 3rd
Generation Partnership Project. https://www.etsi.org/deliver/etsi\protect\T1\textdollar_\protect\T1\textdollartr/138900\protect\T1\textdollar_\protect\T1\textdollar138999/138901/16.01.00\protect\T1\textdollar_\protect\T1\textdollar60/tr\protect\T1\textdollar_\protect\T1\textdollar138901v160100p.pdf.

